# Phase II feasibility study of adjuvant chemotherapy with docetaxel/cisplatin/S-1 followed by S-1 for stage III gastric cancer

**DOI:** 10.1186/s12885-021-08795-4

**Published:** 2021-10-01

**Authors:** Noriyuki Hirahara, Takeshi Matsubara, Shunsuke Kaji, Tetsu Yamamoto, Ryoji Hyakudomi, Kiyoe Takai, Kazunari Ishitobi, Yuki Uchida, Yoshitsugu Tajima

**Affiliations:** Department of Digestive and General Surgery, Shimane University Faculty of Medicine, 89-1 Enya-cho, Izumo, Shimane 693-8501 Japan

**Keywords:** Docetaxel/cisplatin/S-1, Gastric cancer, Adjuvant chemotherapy, R0 resection

## Abstract

**Background:**

This study aimed to evaluate the feasibility, safety, and efficacy of postoperative adjuvant chemotherapy with docetaxel/cisplatin/S-1 (DCS) following S-1 therapy in patients with stage III gastric cancer after curative gastrectomy.

**Methods:**

Patients with stage III gastric cancer who underwent D2 gastrectomy were enrolled. Adjuvant chemotherapy was initiated within 8 weeks of gastrectomy. The first cycle of chemotherapy consisted of S-1 monotherapy (day 1–14), followed by a 7-day rest period. Cycles 2 and 3 consisted of the following: S-1 (day 1–14) administration, followed by a 14-day rest period, and an intravenous infusion of cisplatin and docetaxel on days 1 and 15. After two cycles, S-1 was administered for up to 1 year.

**Results:**

Thirty patients were enrolled between 2014 and 2017. Febrile neutropenia of grade 3 or higher was the most common hematological toxicity with 4 patients (13.3%). Other hematological toxicities of grade 3 or higher were as follows: neutropenia in 3 (10.0%), leukopenia in 3 (10.0%), and anemia in 2 (6.7%) patients. Most frequent non-hematological toxicity of grade 3 was anorexia (*n* = 4, 13.3%) and general fatigue (*n* = 3, 10.0%); no grade 4 non-hematological toxicities were observed. Twenty-five patients (83.3%) completed two cycles of DCS treatment and 18 (60.0%) completed subsequent S-1 treatment for 1 year. The relative dose intensity of docetaxel and cisplatin was 0.86 and that of S-1 was 0.88.

**Conclusion:**

The DCS regimen can be acceptable as an adjuvant chemotherapy and offers an effective postoperative treatment option for stage III gastric cancer patients.

**Trial registration number:**

UMIN000012785.

**Date of registry:**

08/01/2014.

## Background

Postoperative adjuvant chemotherapy for gastric cancer has been used to eradicate minimal residual disease. In particular, patients with advanced gastric cancer, who are at a high risk of recurrence even after curative resection, require effective postoperative adjuvant chemotherapy to prolong survival. In 2006, the Adjuvant Chemotherapy Trial of TS-1 for Gastric Cancer (ACTS-GC), which was a phase III trial, conducted a two-arm study comparing the effects of 1-year adjuvant chemotherapy with S-1 and surgery alone, after curative gastrectomy for stage II or III gastric cancer. This study demonstrated a survival benefit of adjuvant chemotherapy with S-1; the 5-year survival rate was 71.7% in the S-1 group and 61.1% in the surgery alone group, with a hazard ratio (HR) of 0.669 (95% confidence interval [CI]: 0.54–0.828) [1]. Based on these results, adjuvant treatment with S-1 has become the standard treatment for stage II/III gastric cancer patients who have undergone radical surgery, according to the 13th edition of the General Rules for Gastric Cancer Treatment [2]. Meanwhile, a subgroup analysis for stage IIIB disease showed that patients in the S-1 group were associated with better overall survival (OS) than patients who underwent surgery alone, regardless of sex, age, or histology, with an HR of 0.791 (95% CI: 0.52–1.205); however, there was no statistically significant survival benefit [3]. These results may be affected by the limitations in the number of patients enrolled in the subgroup analysis; therefore, further improvement in adjuvant chemotherapy for stage IIIB gastric cancer patients is needed. Many exploratory clinical trials and several phase III studies have been conducted in Japan and overseas to clarify the safety, feasibility, and efficacy of the postoperative administration of S-1 in combination with other anti-cancer agents for advanced gastric cancer [4–6]. However, compliance with adjuvant chemotherapy is challenging because of postoperative physical decline in patients [7, 8]. Various phase II and phase III trials of preoperative chemotherapy are currently ongoing with the aim of improving the treatment outcome of gastric cancer; however, no favorable results with high-quality evidence have been reported [9, 10]. It is very important to include adjuvant chemotherapy as the experimental arm of clinical trials to improve the survival of patients with stage IIIA, IIIB, and IIIC gastric cancer.

In this study, we conducted a phase II trial to evaluate the feasibility, safety, and efficacy of postoperative adjuvant therapy with docetaxel/cisplatin/S-1 (DCS) following S-1 therapy in patients with stage IIIA, IIIB, and IIIC gastric cancer after R0 gastrectomy.

## Methods

### Inclusion and exclusion criteria

Patients who met the following eligibility criteria were enrolled in this study: (1) histologically confirmed stage IIIA, IIIB, or IIIC gastric cancer [11]; (2) patients receiving curative gastrectomy with a D2 lymph node dissection or greater [12]; (3) macroscopic tumor type was neither Borrmann type 4 nor large (≥ 8 cm) type 3; (4) no prior chemotherapy or radiation therapy, including treatment for other types of cancer; (5) performance status of 0–1 on Eastern Cooperative Oncology Group classification; (6) no gastric stump cancer; (7) no esophageal invasion or invasion of ≤3 cm; (8) able to receive chemotherapy within 4–8 weeks after surgery; (9) aged between 25 and 75 years; (10) sufficient oral intake; (11) adequate organ function (white blood cell [WBC] count ≥3500/mm^3^ and < 12,000/mm^3^, neutrophil count ≥2000/mm^3^, hemoglobin level ≥ 9.0 g/dl, platelet count ≥100,000/mm^3^, total bilirubin level ≤ 1.5 mg/dl, aspartate transaminase (AST), and alanine aminotransferase (ALT) levels ≤100 IU/l; (12) creatinine level ≤ 1.2 mg/dl, creatinine clearance > 60 ml/min in the Cockcroft-Gault equation; and (13) written informed consent provided by the patient to participate in the study.

The following patients were excluded: (1) synchronous or metachronous (within 5 years) duplication cancers other than carcinoma in situ; (2) contraindications to S-1, cisplatin, and docetaxel; (3) current treatment with systemic steroids; (4) continued use of flucytosine, phenytoin, or warfarin; (5) a history of serious drug hypersensitivity; (6) serious comorbidities (intestinal paralysis, intestinal obstruction, interstitial pneumonia or pulmonary fibrosis, neuropathy, uncontrolled diabetes, heart failure, renal failure, or hepatic failure, etc.); (7) severe mental disorders; (8) history of myocardial infarction or unstable angina pectoris within 6 months; (9) diarrhea (watery stool); (10) pregnancy or breastfeeding; (11) hepatitis B surface antigen positivity; and (12) those deemed ineligible for this study by the investigator or sub-investigator.

This study was approved by the Shimane University Institutional Committee on Ethics and was conducted in accordance with the Declaration of Helsinki and the Japanese Ethical Guidelines for Clinical Studies. This study was registered with the University Hospital Medical Information Network Clinical Trials Registry, number 000012785. Written informed consent was obtained from the patients prior to the study.

### Treatment schedule

Adjuvant chemotherapy was initiated 4–8 weeks after the gastrectomy. The first cycle of chemotherapy consisted of S-1 monotherapy, which was administered twice daily at the following oral doses based on the patient’s body surface area (BSA): 40 mg (BSA < 1.25 m^2^), 50 mg (BSA ≥ 1.25 m^2^), or 60 mg (BSA ≥ 1.5 m^2^), from day 1 to day 14, followed by a 7-day rest. Cycles 2 and 3 adhered to the following schedule: oral administration of S-1 (same dose as that of the first cycle) twice daily from day 1 to day 14, a 14-day rest, followed by intravenous infusion of cisplatin at 35 mg/m^2^ for 2 h and intravenous infusion of docetaxel at 35 mg/m^2^ for 2 h on days 1 and 15. After two cycles, S-1 was administered for up to 1 year. To avoid cisplatin-induced renal dysfunction, adequate hydration with normal saline (> 2000 ml) was administered on days 1 and 15.

Antiemetic analgesics were routinely prescribed to prevent nausea and vomiting. On day 1, all patients received aprepitant 125 mg orally 60 min before cisplatin infusion plus intravenous palonosetron (0.75 mg) and dexamethasone (12 mg) approximately 30 min prior to receiving cisplatin. On days 2 and 3, all patients received oral aprepitant 80 mg once daily after breakfast. Rescue antiemetics, such as 5-hydroxy-tryptamine-3 (5-HT_3_) receptor antagonists, were prescribed to treat prominent, unbearable nausea and vomiting. These prophylactic antiemetics were used in accordance with the Japan Society of Clinical Oncology Guidelines for Antiemetics in Oncology 2010 [13].

### Toxicity assessment

The patient’s symptoms and physical examination results were obtained. The assessment and grading of blood test were done according to the National Cancer Institute Common Toxicity Criteria for Adverse Events version 4.0 (https://ctep.cancer.gov/protocoldevelopment/electronic_applications/ctc.htm#ctc_40).

Subsequent chemotherapy was postponed if a patient did not meet the following criteria: WBC count ≥3000 mm^3^, neutrophil count ≥1500 mm^3^, hemoglobin level ≥ 8.0 g/dl, platelet count ≥75,000 mm^3^, total bilirubin level ≤ 1.5 mg/dl, AST and ALT levels ≤100 IU/l, and creatinine concentration < 1.2 mg/dl. After confirming non-hematological toxicities, body temperature, general fatigue, anorexia, stomatitis, diarrhea, watery eyes, and neuropathy were graded (0 or 1) for adjuvant chemotherapy, and other non-hematological toxicities were graded as 2 or lower.

The dose of S-1 was reduced by 20 mg/day when any adverse events mentioned above were observed during the previous cycle. Patients in whom the dose of S-1 was already reduced to 80 mg per day were withdrawn from the study when adverse events reoccurred. The doses of docetaxel and cisplatin were reduced from 35 to 20 mg/m^2^ in case of any adverse events. Moreover, if patients could not resume subsequent cycles within 4 weeks due to toxicities, they were withdrawn from the study.

### Follow-up

During the study, physical examination, complete blood cell counts, and biochemical examination were performed on the first day of each chemotherapy cycle, and every 3 months thereafter. Computed tomography was performed every 6 months for the first 3 years, and yearly thereafter.

### Statistical analysis

The primary endpoint of this study was the completion rate of two courses of DCS chemotherapy. Secondary endpoints were OS, recurrence-free survival (RFS), cumulative 1-year S-1 completion rate, and the safety of the chemotherapy regimen. OS was defined as the time from registration to the date of death from any cause. RFS was defined as the interval from registration until objective tumor recurrence. OS and PFS were estimated using the Kaplan-Meier method. Data for patients who remained event-free at clinical time were censored on the day they were last reviewed. Relative dose intensity (RDI) was defined as the administered dose divided by the planned dose.

Although it was difficult to establish the number of patients based on statistical hypothesis since this was an exploratory study, we assumed the expected rate of completion of two courses of S-1 with DCS therapy in 30 patients to be 60% (18 patients) and the 95% CI to be 0.425–0.775, with an estimate CI range of 0.351.

All statistical analyses were conducted using JMP software for Windows (version 14.0; SAS Institute, Cary, NC, USA).

## Results

### Patient characteristics

Thirty patients were enrolled in this study between January 2014 and December 2017. The characteristics of the patients are summarized in Table 1. There were 26 men and 4 women, with a median age of 69 years (range, 51–79 years). Total gastrectomy, proximal gastrectomy, and distal gastrectomy were performed in 12 patients (40%), 2 patients (6.7%), and 16 patients (53.3%), respectively. Histologically, 2 patients (6.7%) had well-differentiated adenocarcinoma, 12 (40%) had moderately differentiated adenocarcinoma, and 16 (53.3%) patients had undifferentiated adenocarcinoma. In regard to cancer staging, 12 patients (40.0%) had stage IIIA, 9 patients (30.0%) had stage IIIB, and 9 patients (30.0%) had stage IIIC cancer.

**Table 1:** **Baseline patient characteristics**

Characteristics	No. of patients (%)	
Median age year, (range)	69	(51–79)
Gender		
male	26	(86.7)
female	4	(13.4)
Median BMI, (range)	22.2	(15.3–28.5)
Primary lesion		
Upper	8	(26.7)
Middle	11	(36.7)
Lower	11	(36.7)
Type of gastrectomy		
Proximal	2	(6.7)
Distal	16	(53.3)
Total	12	(40.0)
Macroscopic type		
1	1	(3.3)
2	5	(16.7)
3	22	(73.3)
5	2	(6.7)
Differentiation		
well	2	(6.7)
mod	12	(40.0)
poor	16	(53.3)
Tumor stage		
3	14	(46.7)
4a	16	(53.3)
Nodal stage		
1	2	(6.7)
2	13	(43.3)
3a	12	(40.0)
3b	3	(10.0)
UICC stage		
IIIA	12	(40.0)
IIIB	9	(30.0)
IIIC	9	(30.0)

### Toxicity

Table 2 summarizes the adverse events observed in patients administered adjuvant chemotherapy with DCS. Febrile neutropenia of grade 3 or higher was the most common hematological toxicity which was associated with 4 patients (13.3%). Other hematological toxicities, which were grade 3 or higher, were as follows: neutropenia in 3 patients (10.0%), leukopenia in 3 patients (10.0%), and anemia in 2 patients (6.7%). The most frequent non-hematological toxicity of grade 3 or higher was anorexia, which was observed in 4 patients (13.3%), followed by general fatigue in 3 patients (10.0%).

**Table 2:** Adverse events observed in patients administered adjuvant chemotherapy with DCS

Toxicity	No. of patients (%)			
	Grade 3 or higher		All	
Hematological toxicity				
Neutropenia	3	(10.0)	14	(46.7)
Leukopenia	3	(10.0)	12	(40.0)
Anemia	2	(6.7)	10	(33.3)
Thrombocytopenia	0	(0.0)	8	(26.7)
Febrile neutropenia	4	(13.3)	4	(13.3)
Non-hematological toxicity				
Nausea	1	(3.3)	19	(63.3)
Diarrhea	1	(3.3)	6	(20.0)
Anorexia	4	(13.3)	22	(73.3)
Fatigue	3	(10.0)	23	(76.7)
Stomatitis	0	(0.0)	5	(16.7)
Abdominal distension	0	(0.0)	2	(6.7)
Alopecia	0	(0.0)	17	(56.7)
Dry skin	0	(0.0)	2	(6.7)
Dysgeusia	0	(0.0)	3	(10.0)
Skin pigmentation	0	(0.0)	4	(13.3)

Table 3 summarizes the adverse events observed in patients administered S-1 adjuvant chemotherapy. Grade 3 or higher adverse events were neutropenia, leukopenia, nausea, diarrhea, and anorexia, which were observed in one case each.

**Table 3:** Adverse events observed in patients administered adjuvant chemotherapy with S-1

Toxicity	No. of patients (%)			
	Grade 3 or higher		All	
Hematological toxicity				
Neutropenia	1	(3.3)	9	(30.0)
Leukopenia	1	(3.3)	8	(26.7)
Anemia	0	(0.0)	5	(16.7)
Thrombocytopenia	0	0.0	3	(10.0)
Febrile neutropenia	0	(0.0)	1	(3.3)
Non-hematological toxicity				
Nausea	1	(3.3)	8	(26.7)
Diarrhea	1	(3.3)	4	(13.3)
Anorexia	1	(3.3)	12	(40.0)
Fatigue	0	(0.0)	11	(36.7)
Stomatitis	0	0.0	4	(13.3)
Abdominal distension	0	0.0	1	(3.3)
Alopecia	0	0.0	1	(3.3)
Dry skin	0	0.0	3	(6.7)
Dysgeusia	0	0.0	4	(13.3)
Skin pigmentation	0	0.0	6	(20.0)

Granulocyte-colony-stimulating factor (G-CSF) and antibiotics were required for 4 patients who had febrile neutropenia of grade 3 or higher. All chemotherapy-related toxicities were resolved with appropriate care, and treatment-related deaths were not observed in this study. Non-hematological toxicities were generally mild, and grade 4 patients were not observed.

### Treatment compliance

Table 4 summarizes treatment compliance in this study. Twenty-five patients (83.3%) completed two cycles of the DCS treatment, and 18 patients (60.0%) completed subsequent S-1 treatment for 1 year. Dose reduction of DCS was required in 6 patients (20.0%). The second DCS cycle was delayed in 4 patients owing to several reasons, such as general fatigue (*n* = 2, 6.7%) and anorexia (n = 2, 6.7%). The dose of S-1 was reduced in 6 patients (20.0%), mainly due to a grade 3 or higher neutropenia (n = 2, 6.7%) and anorexia (*n* = 4, 13.3%).

**Table 4:** Compliance to adjuvant chemotherapy

	No. of patients (%)	
Cycle of DCS		
1	29	(96.7)
2	25	(83.3)
S-1 administration period (month)		
3	28	(93.3)
6	27	(90.0)
9	23	(76.7)
12	18	(60.0)

The RDI of docetaxel and cisplatin was 0.86, and the RDI of S-1 for the DCS treatment was 0.88.

One patient withdrew from the study because the patient refused subsequent chemotherapy due to disagreeable toxicities.

### Survival analysis

The median follow-up period was 1335 days (range, 684–2639). All patients were followed-up for at least 3 years from the date of registration. At the time of the date cut-off on December 31, 2020, 10 patients had died of primary disease, 2 patients survived with recurrence, and the remaining 18 remained well without recurrence.

**Fig. 1:**
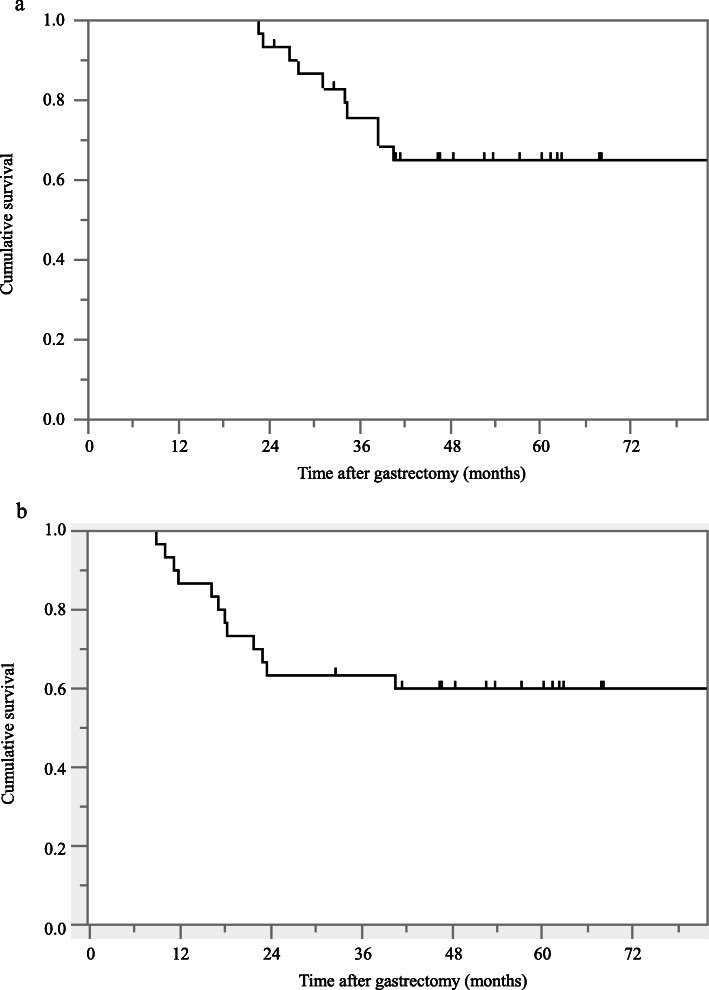
The Kaplan-Meier estimates indicate overall survival (**a**) and relapse-free survival (**b**) after curative gastrectomy for stage III gastric cancer

The 1-, 2-, and 3-year OS rates were 100, 93% (95% CI: 79.8–99.5), and 72.1% (95% CI: 53.3–85.4), respectively (Fig. 1a). The 1-, 2-, and 3-year RFS rates were 86.7% (95% CI: 69.4–94.9), 66.7% (95% CI: 48.3–81.0), and 63.3% (95% CI: 45.1–78.3), respectively (Fig. 1b).

## Discussion

In the present study, we investigated whether the DCS chemotherapy regimen is safe to be administered as postoperative adjuvant therapy with acceptable toxicity in patients who underwent R0 resection for gastric cancer. Adjuvant chemotherapy after curative surgery is beneficial in reducing recurrence and improving OS in patients with resectable gastric cancer [1, 14, 15]. However, strict consideration should be given to tolerability of patients and safety of the regimen when administering multidrug adjuvant chemotherapy since postoperative patients are often physically weak, [16–18] and gastrectomy-induced decline in tolerability to chemotherapy may limit intensive adjuvant treatment. Therefore, attention towards neoadjuvant chemotherapy (NAC) is increasing; however, effective NAC regimens with high-quality evidence are yet to be developed [19, 20]. Further refinement and modification of postoperative regimens without compromising the patient’s quality of life is important to improve the clinical outcome of patients with gastric cancer.

In Japan, the standard regimens considered as adjuvant chemotherapy for gastric cancer are S-1 for pathological stage II and docetaxel plus S-1 for stage III patients, based on the results of the ACTS-GC [1, 3] and START-II trials [20], respectively. The ACTS-GC trial revealed that 66.4% of the enrolled patients were tolerable to S-1 alone for 1 year. While the START-II trial was conducted to prove the superiority of administering postoperative S-1 plus docetaxel over S-1 alone for pathological stage III gastric cancer patients; it revealed that 234 (69%) of the 320 patients tolerated the originally planned 6 doses of docetaxel treatment, with a dose reduction in 94 patients (28%). Regarding patient compliance with S-1, administration of S-1 for 1 year persisted in 168 patients (49%) in the S-1 plus docetaxel group and 195 patients (56%) in the S-1 alone group.

A phase I study including patients with unresectable metastatic gastric cancer at the Sapporo Medical University determined the recommended doses (RDs) to be as follows: 60 docetaxel 80 mg/m^2^ (day 8), cisplatin 60 mg/m^2^ (day 8), and S-1 80 mg/m^2^ (day 1–14) for a 3-week course. A subsequent phase II study reported an extremely high response rate of 87.1% [21, 22]. In a phase I study for patients with unresectable or recurrent gastric cancer, Kitasato University reported the RDs to be as follows: docetaxel 40 mg/m^2^ (day 1), cisplatin 70 mg/m^2^ (day 1), and S-1 80 mg/m^2^ (day 1–14) for a 4-week course [23]. In a subsequent phase II study, although the dose of cisplatin was reduced to 60 mg/m^2^ owing to myelosuppression and renal damage, the overall response rate was as high as 81.3% [24]. On the other hand, Kanazawa University reported a phase I study in which docetaxel and cisplatin were administered as preoperative chemotherapy at 2-week intervals (days 1 and 15). docetaxel 35 mg/m^2^ (days 1 and 15), cisplatin 35 mg/m^2^ (days 1 and 15), and S-1 80 mg/m^2^ (days 1–14) were considered as the RD in a 4-week course and the response rate was 77.8% [25]. While the Sapporo Medical University regimen is expected to have a high response rate, the frequency of serious adverse events is also high with grade 3 or 4 neutropenia (77.4%) and febrile neutropenia (16.1%), and it is considered as an inappropriate regimen for unresectable or recurrent gastric cancer that cannot be cured. In contrast to this, the Kitasato University regimen demonstrated significant adverse events (grade 3 or 4 neutropenia: 72.8%, febrile neutropenia: 13.5%) in all phase II studies. The Kanazawa University regimen is expected to be used for postoperative adjuvant chemotherapy because it aims to reduce adverse events by administering docetaxel and cisplatin in divided doses. This suggests that the Kanazawa University regimen may reduce adverse events without decreasing efficacy. Based on the above factors, the Kanazawa University regimen was selected as the treatment in this study.

As with preoperative chemotherapy, there is no clear rationale for the number of courses of postoperative adjuvant therapy. Takahari et al. [26] reported that three cycles of S-1 plus cisplatin was feasible as adjuvant chemotherapy and provided a survival benefit to patients with stage III gastric cancer. Since DCS is more toxic, we assumed that it would be less tolerable, and therefore, we limited the number of courses. If the results of this study are favorable, it may be possible to increase the number of cycles to enhance its anti-tumor effects.

In the present study, a relatively high tolerance for DCS treatment was also observed, i.e., 25 (83.3%) patients completed two courses of DCS and 18 (60.0%) patients subsequently received S-1 for 1 year; this was probably because docetaxel and cisplatin were administered biweekly in two divided doses, after anticipating a decrease in toxicity [21–25]. Takahari et al. [26] investigated the tolerability of adjuvant therapy with S-1 plus cisplatin in patients with stage III gastric cancer who underwent gastrectomy, based on the original protocol stating three courses of S-1 plus cisplatin followed by S-1 alone for 1 year after surgery. However, the protocol was revised due to a low completion rate of the study that was revealed in the interim evaluation; following which, S-1 alone was the first course and S-1 plus cisplatin were courses 2–4, finally followed by S-1 alone until 1 year after surgery. As a result, the completion rates of 3 courses of S-1 plus cisplatin therapy improved from 57% before revision to 81% after revision [26]. The S-1 plus cisplatin regimen was safer and better tolerated when cisplatin was administered after the second course of S-1, than administering cisplatin in the first course. In the present study, S-1 monotherapy was selected as the first course.

Sato et al. [22] and Nakayama et al. [24] reported that grade 3 and grade 4 neutropenia was observed in 77.4 and 42.9% of patients after administering DCS, respectively. In our study, febrile neutropenia was the most common hematological toxicity, accounting for 13.3% of cases. In addition, patients with grade 4 neutropenia or grade 3 or 4 febrile neutropenia required the use of G-CSF, with a short duration of neutropenia. In patients who required G-CSF in the first cycle, the occurrence of neutropenia and febrile neutropenia was reduced in the second cycle because G-CSF was administered prophylactically to these patients. The most common grade 3 non-hematologic toxicities in our study were anorexia (13.3%) and general fatigue (10.0%). Grade 3 nausea was observed in only 3.3% of patients. Anorexia and nausea were usually tolerable and managed with a planned protocol of prophylactic administration of antiemetics (5-HT3 antagonists plus corticosteroids) during cisplatin administration. Notably, adjuvant chemotherapy was continued according to the protocol in most cases. In addition, most toxicities associated with chemotherapy were resolved by prolonging the onset of treatment, reducing the dose of anti-cancer drugs, or providing appropriate supportive care. As a result, there were no treatment-related deaths in this study.

With regard to the survival analysis, the 1-, 2-, and 3-year OS rates were 100, 93, and 72.1%, respectively (Fig. 1a), and the 1-, 2-, and 3-year RFS rates were 86.7, 66.7% (95% CI: 48.3–81.0), and 63.3%, respectively. The present study indicated survival benefits for patients with stage III gastric cancer despite a small number of cases and a relatively short observation period.

Future directions for chemotherapeutic research include administering oxaliplatin, a platinum-based drug with reduced toxicity, for the gastrointestinal system and kidney. In addition, intensive management of chemotherapy-related adverse effects with prophylactic agents is necessary. Olanzapine is an atypical antipsychotic drug that inhibits neurotransmitter pathways known to be involved in nausea and vomiting [27, 28]. Evaluation of the efficacy of olanzapine in combination with standard antiemetic therapy for the prevention of chemotherapy-induced nausea and vomiting is warranted.

Although our study provided some useful information, several limitations need to be considered when interpreting the results of this study. First, our clinical trial may have enrolled relatively healthy patients, that is, there may be a selection bias related to performance status, age, and comorbidities. Second, the study was not sufficiently powered to identify specific populations that are more likely to derive benefits from DCS chemotherapy because of its small sample size. Third, we initially planned to recruit 10 patients per year; however, there were not enough patients diagnosed with stage III gastric cancer due to an unexpectedly low incidence of gastric cancer in Japan, which is probably due to *Helicobacter pylori* eradication and the widespread use of gastric cancer screening [29, 30]. Therefore, the study required a longer time to enroll eligible patients, and the chemotherapy regimen recommended by the Japanese Guidelines for the Treatment of Gastric Cancer was revised during the study period [31].

## Conclusions

Our phase II study revealed that the DCS regimen can be acceptable as adjuvant chemotherapy in terms of safety and toxicity, and may offer a viable postoperative treatment option for patients with stage III gastric cancer.

## Data Availability

The datasets used and analyzed during the current study are available from the corresponding author on reasonable request.

## Abbreviations

5-HT3: 5-hydroxy-tryptamine-3
ACTS-GC: Adjuvant Chemotherapy Trial of TS-1 for Gastric Cancer
ALT: alanine aminotransferase
AST: aspartate transaminase
BSA: body surface area
CI: confidence interval
DCS: docetaxel/cisplatin/S-1
G-CSF: granulocyte-colony-stimulating factor
HR: hazard ratio
NAC: neoadjuvant chemotherapy
OS: overall survival
RD: recommended dose
RDI: relative dose intensity
RFS: recurrence-free survival
WBC: white blood cell
